# Electroactive Textile Actuators for Breathability Control and Thermal Regulation Devices

**DOI:** 10.3390/polym11071199

**Published:** 2019-07-18

**Authors:** Chaoqun Xiang, Jianglong Guo, Rujie Sun, Andrew Hinitt, Tim Helps, Majid Taghavi, Jonathan Rossiter

**Affiliations:** 1 SoftLab, Bristol Robotics Laboratory, University of Bristol, Bristol BS16 1QY, UK; 2 Bristol Composites Institute (ACCIS), University of Bristol, Bristol BS8 1TR, UK

**Keywords:** breathability, conductive textile, dielectric elastomer actuator, wearable device

## Abstract

Smart fabrics offer the potential for a new generation of soft robotics and wearable technologies through the fusion of smart materials, textiles and electrical circuitries. Conductive and stretchable textiles have inherent compliance and low resistance that are suitable for driving artificial muscle actuators and are potentially safer electrode materials for soft actuation technologies. We demonstrate how soft electroactive actuating structures can be designed and fabricated from conducting textiles. We first quantitatively analyse a range of stretchable conductive textiles for dielectric elastomer actuators (DEAs). We found that conductive-knit textiles are more suitable for unidirectional DEA applications due to the largest difference (150%) in principle strain axes, whereas isotropic textiles are more suited to bidirectional DEA applications due to the smallest (11.1%) principle strain difference. Finally, we demonstrate controllable breathability through a planar e-textile DEA-driven skin and show thermal regulation in a wearable prototype that exploits soft actuation and kirigami.

## 1. Introduction

Conventional robots are predominantly made of rigid structural materials such as metals or plastics [1,2]. They are extremely effective and powerful in various manufacturing and industrial automation applications involving labour-intensive tasks such as material handling in assembly lines [1]. Rigid robots are heavy and powered by energy-intensive electrical motors and solenoids or high pressure pneumatically and hydraulically pressurized fluids [1,2]. In addition, hard robots are often biologically or biomechanically incompatible [3]. To interact with humans, these rigid robots require advanced sensing and control systems, significantly increasing cost and complexity [3]. Soft robots, in contrast, are made of intrinsically soft and stretchable materials such as gels and elastomers, and are formed into compliant active mechanisms and structures [1,2,3,4,5,6,7]. They are, therefore, able to offer safer and more robust interactions with humans and natural environments. In addition, they are resilient and capable of actively and passively changing their shapes to adapt to unpredictable and unstructured environments through simple, often morphologically embedded, control mechanisms. Soft robotics, as an emerging research field, is exploring new robotic capabilities such as active morphing [8] and self-healing [9] that conventional robots cannot achieve. Soft functional devices can enable a new generation of much safer, robust and reliable soft-soft interactions between implanted and wearable devices and living tissues [10].

The on-going development of soft robotics has increased the need for better performing smart and soft materials and transducers. Soft transducers include soft sensors, actuators and generators. Soft actuators are components that convert, by electric power, low pressure hydraulic fluid or pneumatic pressure into mechanical movements. Advances in soft actuating technologies are critically required to promote the paradigm shift from conventional robotics to soft robotics.

Dielectric elastomer actuators (DEAs), a major artificial muscle technology, are made of thin elastomeric membranes sandwiched between stretchable and compliant electrodes [9]. When an electric field (usually in the range of MV/m) is applied, induced Maxwell pressure between the two electrodes deforms the structure, resulting in electrode area expansion and membrane thickness reduction. DEAs are a promising and popular soft actuation technology because: (1) they can produce large deformations, (2) their actuation responses are fast, (3) they have high energy density and (4) they are lightweight and cost-effective [11,12]. Because of these advantages, DEAs have been extensively studied in the last two decades and demonstrated in various proof-of-concept devices including lenses [13], grippers [8] and robots [11,12,13,14].

Compliant, low resistance, soft and stretchable electrodes are an important component not only for DEA technologies but also for the wider flexible and stretchable electronics field. Liquid metal electrodes are inherently compliant, low resistance, soft and stretchable, but require complex encapsulation technologies [15]. Hydrogel electrodes must retain their moisture content, which significantly limits their applications, and their ionic conductivity can result in unwanted chemical reactions and electrolysis depending on the voltages and currents employed [16]. Stretchable thin film metal electrodes have low resistance but need to be manufactured using expensive deposition, sputtering, evaporation or photolithographic processes [17]. Carbon is the most common electrode material in DEAs since it is low cost and easy to use. Unfortunately, compliant carbon electrodes have relatively high resistance (usually above 10 kΩ), which increases the device time constant, limits dynamic response and reduces energy efficiency. Carbon electrodes are typically fabricated by mixing oil with carbon powders to make carbon grease, or by mixing carbon black, graphite, graphene or carbon nanotubes with curable soft elastomers. Carbon grease has been the prevailing electrode materials for DEA applications since it is low cost and easy to procure and use. Carbon grease cannot, however, be cured at room temperature, and it is messy and potentially carcinogenic. There is, therefore, a need for a safe, compliant, low resistance, soft, and stretchable DEA electrode material and a simple, clean and easy-to-implement electrode fabrication method.

Conductive textiles are stretchable, have low resistance [17,18] and are potentially safer electrode materials [19]. The fabrication of conductive textile electrodes is simple and straightforward [19,20], and they have been presented as potential electrode materials for DEAs and soft EA applications [19,20,21]. In [19], Guo at al. used a low cost two-dimensional (2D) desktop material cutter to fabricate textile electrodes for simple soft DEA and electroadhesive (EA) actuators. These included tubular DEAs, soft EAs and an entirely textile driven DEA-EA soft crawler. In [20], Allen et al. compared two conductive textiles and used them (using a laser cutter to cut the electrodes) in a variable stiffness DEA device. Both studies manifest that conductive and stretchable textiles have the potential to enable simple, comfortable and wearable soft robotic devices and complete robots. In this paper, we examine and compare five different conductive and stretchable textiles for DEA applications, as presented in Section 2. For future smart clothes it will be important to have active breathability control capabilities, especially where the body sweats in response to changes in ambient heat and humidity and as we exercise and generate in-body heat. In addition to breathability, we require smart clothing to react to changes in environmental temperature. In Section 3, we will explore the use of e-textile DEAs, combined with kirigami structures, for the development and characterization of a breathability control device and a thermal regulation device.

## 2. Conductive Textile Dielectric Elastomer Actuation

We selected five readily available conductive and stretchable textiles: (1) EeonTex, (2) Softmesh, (3) Knit Jersey, (4) Conductive-knit Fabric and (5) ElectroLycra. We used a Cricut 2D computer-controlled material cutter (Provo Craft & Novelty, Inc., South Jordan, UT, USA) to cut these textiles into 40 mm × 40 mm square sheets. We measured their mass and thickness and measured end-to-end resistance using a digital multimeter (AM-500-EUR, RS Components, Bristol, UK), waiting 30 s to stabilize the resistance reading. Three tests were repeated for each textile. The mean values and one standard deviation are presented in Table 1.

Using the five textiles shown in Table 1, we manufactured DEAs based on the following four steps: 1. We laser cut five 5 mm thick circular acrylic plates with an inner diameter of 120 mm and an outer diameter of 130 mm; 2. We cut (using the Cricut 2D cutter) two circular conducting textile electrodes with a diameter of 24 mm and a single connection tab of 6 mm width. Ten plastic masks of the same size were also prepared, providing registration for conductive textile bonding in step 4 and ensuring that the textile electrodes were aligned in the centre of the actuator; 3. We pre-stretched five 1 mm thick VHB 4910 dielectric membranes (3M, Maplewood, MN, USA) by a biaxial stretcher from diameter 35 to 145 mm, yielding 414% linear pre-strain and 1700% area pre-strain and a final membrane thickness of 59 μm; 4. We then temporarily attached the masks onto the membranes and bonded the circular textile electrodes onto the centre of the membrane (due to the intrinsic adhesion of the VHB film) and finally removed the masks, forming five DEAs.

**Figure 1:**
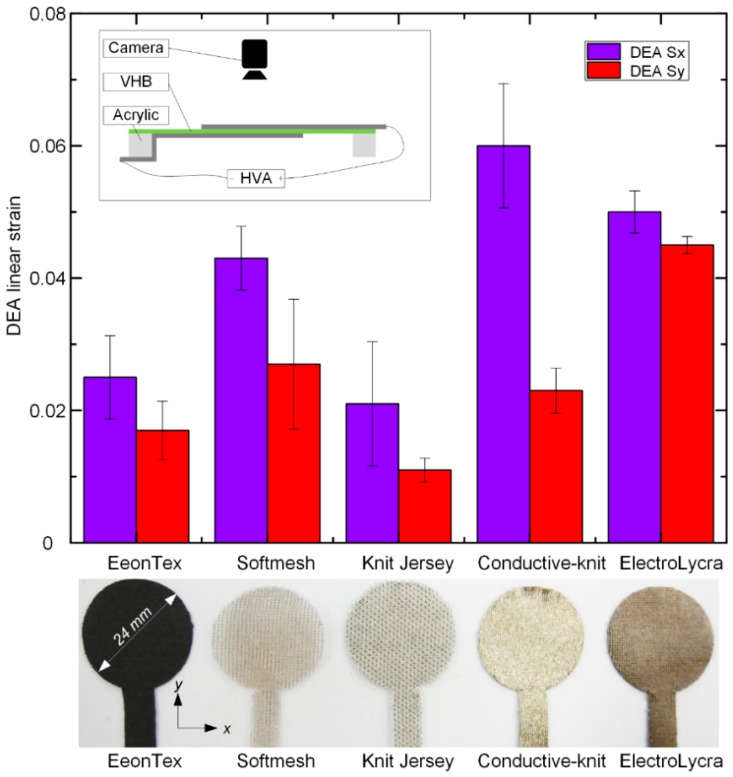
Comparison of dielectric elastomer actuator (DEA) strains in *x* and *y* axis for the five conductive textiles at 6 kV. Inset shows the schematic diagram of the test setup. VHB is a 3M adhesive tape brand and HVA is the abbreviation of high voltage amplifier.

We then applied 6 kV to each DEA using an Ultravolt high voltage amplifier (10HVA24-BP1, Advanced Energy Industries, Inc., Fort Collins, CO, USA, maximum voltage 10 kV) for 10 s. A 50 fps camera (DMC-G80, Panasonic, Berkshire, UK) was used to record the dynamic area change of the DEA electrodes when actuated and the linear strain in the predominant direction (labelled *x* axis) and orthogonal axis (labelled *y* axis). We found that different textiles had different DEA strains and there was a significant difference in the obtained strains in the *x* and *y* axes (labelled S*_x _*and S*_y_*, respectively). Three tests were repeated for each DEA, and mean values and one standard deviations of the five DEA strains were calculated. Results are shown in Figure 1. Conductive-knit has the largest *x* axis strain (S*_x_* = 0.06 ± 0.009), but the difference between the strains in the *x* axis and *y* axis is also the largest (a relative difference of 150%). ElectroLycra has the smallest difference between the strains in the *x* axis and *y* axis (a relative difference of 11.1%). We define the relative difference in the *x* axis as (max_S*_x_*–min_S*_x_*)/min_S*_x_* × 100%, and the relative difference in the *y* axis as (max_S*_y_*–min_S*_y_*)/min_S*_y_* × 100%.

**Figure 2:**
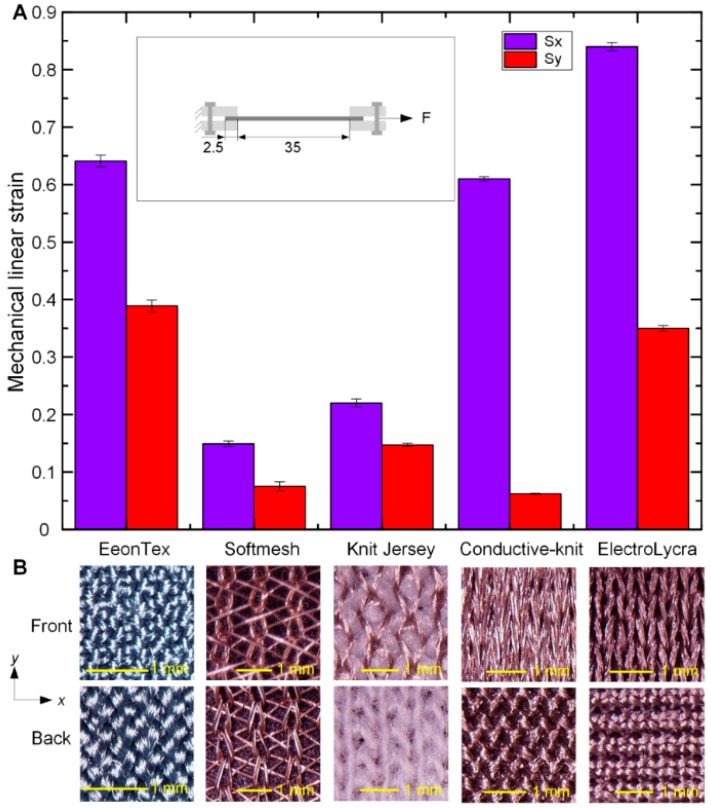
Comparison of the five e-textiles in terms of mechanical strains and microscopic structures. (**A**) Comparison of mechanical strains in *x* and *y* axes for the five conductive textiles under 20 N tensile force. Inset shows the schematic diagram of the test setup. (**B**) Micrograph images of the patterns for each textile from both sides.

We assume that the strain difference is due to the difference in textile structures. To investigate this dependence, we conducted mechanical strain quantification of the five textiles. Square samples (40 mm × 40 mm) were cut using the Cricut, and each sample was clamped at opposite ends using acrylic plates bolted together (extending 2.5 mm in from the edge of the sample). One end was fixed, a constant tensile load of F = 20 N was applied (measured by FG-5000A force gauge, Lutron Electronic Enterprise, Taipei, China) to the other end and linear strains were measured. Three tests were repeated for each e-textile, and the mean values and one standard deviation were calculated. Results are shown in Figure 2. All the textiles were stored together in the same conditions for over 24 h and were used under the same humidity, 51 ± 1%, and temperature, 22.1 ± 0.1 °C. As can be seen in Figure 2A, there is significant anisotropy in the mechanical properties of all the textiles, with the Conductive-knit showing the largest anisotropy and EonTex the least. Figure 2A suggests that Conductive-knit is most effective in unidirectional DEA configurations, while ElectroLycra and EeonTex are more effective for bidirectional (areal) DEAs. Figure 2B also shows optical micrographs (Hirox KH-7700 Digital Microscope System, Tokyo, Japan) of the patterns of each fabric from both sides. For the ElectroLycra textile, micrograph images in Figure 2B show that the knit on one side will limit vertical strain more significantly than horizontal strain, whereas on the other side the knit will result in more uniform strains in both directions. When under the mechanical strain test, a single direction (either vertical or horizontal) of the sample was stretched and this resulted in the largest vertical/horizontal direction strains, whereas in the DEA strain test the material is equi-biaxial stressed and both vertical and horizontal strains interact and limit each other (due to the complex overall structure of the textile), resulting in a smaller discrepancy in the DEA strain results.

**Figure 3:**
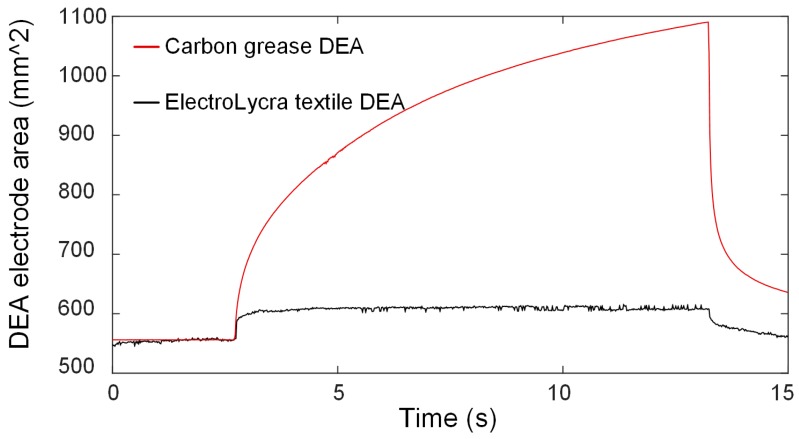
Dynamic DEA electrode area change comparison between carbon grease and ElectroLycra textile DEAs (6 kV applied for 10 s).

We also compared the dynamic electrode area change of the ElectroLycra conductive textile DEA and a common carbon grease (MG Chemicals, Manchester, UK) DEA, as shown in Figure 3. We again applied 6 kV for 10 s to each DEA. The carbon grease DEA generated a higher maximum areal strain (89.2%) than the ElectroLycra conductive textile DEA (6.8%) but suffered from creep under a constant applied voltage. The ElectroLycra, in contrast, reached its maximum strain the fastest and showed minimal creep. Within the 10 s, the carbon grease DEA had not achieved its maximum DEA area strain, whereas the ElectroLycra conductive textile achieved its maximum area strain within 1 s. We propose that this self-limiting action was caused by the stiffening of the textile as it reoriented under actuation and by the uncurling of interlocked warp and weft fibres reaching their limit [22]. At this point no further reorientation is possible and actuation stops.

## 3. DEA Driven Breathability and Thermal Control Devices

We now examine how conductive textiles and their actuators can be used to realise new capabilities in wearable robotics and smart textiles. As new wearables and synthetic materials are made and exploited in smart clothing, we must ensure that they are breathable. Breathability is defined as the ability of a fabric/textile to allow moisture vapour to be transmitted through the material [23]. It is highly desirable to embed active breathability control capabilities into certain parts of our clothing, especially where the body sweats in response to changes in ambient heat and humidity and as we exercise and generate in-body heat. In [24], by coating triacetate-cellulose bimorph fibres with a thin layer of carbon nanotubes and modulating incident infrared radiation, this fabric is shown to automatically cool or insulate depending on conditions. In our research, we propose using textile DEA-driven breathability control devices to enhance smart clothing, and we demonstrate a 2D wearable control device inspired by the active stomata in plant leaves [25] and planar auxetics. In addition to breathability, we require smart clothing to react to temperature changes. Kirigami is the practice of placing strategic cuts in paper, extending the capabilities of origami [26], a term for paper folding. Kirigami can be used to make highly stretchable and morphable two-dimensional (2D) and three-dimensional (3D) structures from non-stretchable 2D materials [27,28]. A novel thermal regulation device that exploits actuation from 2D into 3D using an actuated kirigami structure is demonstrated in Section 3.2. 

### 3.1. 2D Textile Breathability Control Device

**Figure 4:**
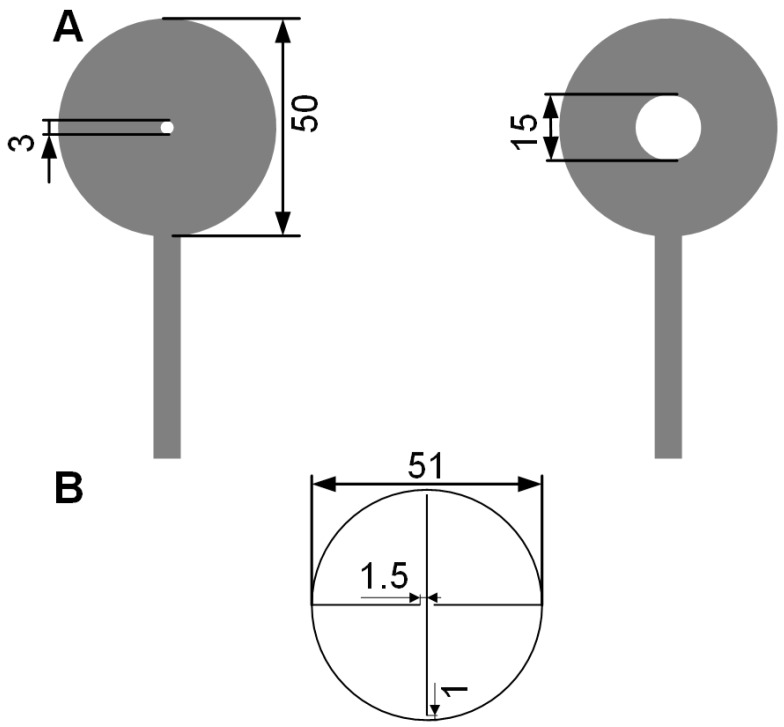
Design parameters for the DEA driven breathability control device: (**A**) Textile electrode design and parameters. (**B**) Circular auxetic structure design and parameters.

**Figure 5:**
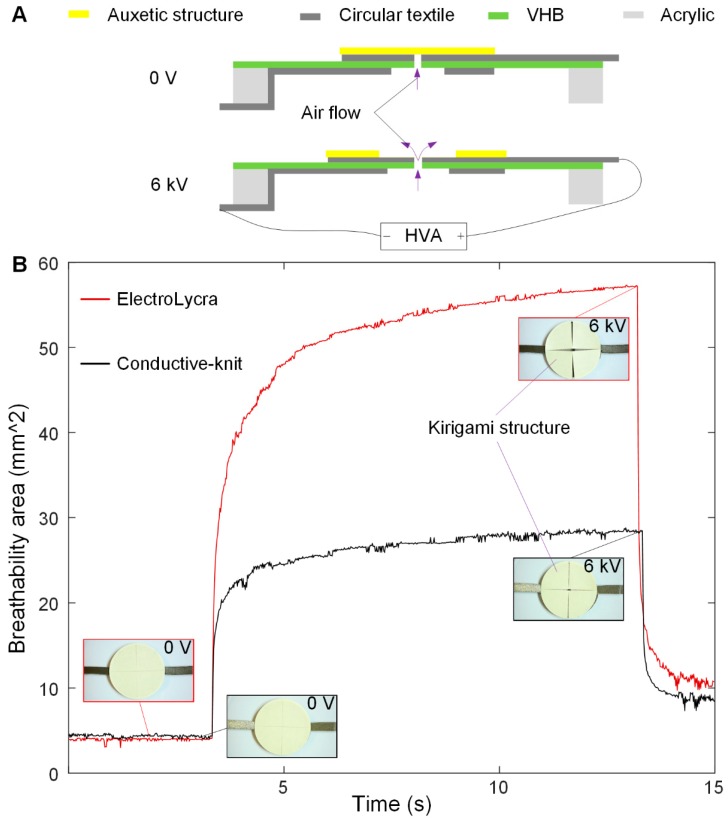
E-textile driven DEA breathability control devices. (**A**) Schematic diagram of the working principle of the device. (**B**) Dynamic breathability area changes of the ElectroLycra and Conductive-knit textile breathability control devices at 6 kV.

We fabricated a 2D textile actuator for breathability control by combining a circular textile DEA with a simple rectilinear auxetic structure. The device fabrication consists of the following four steps: 1. We laser cut a 5 mm thick circular acrylic plate with an inner diameter of 120 mm and an outer diameter of 130 mm. We then pre-stretched a 1 mm thick VHB 4910 dielectric membranes using a biaxial stretcher from diameter 35 to 145 mm and bonded the acrylic plate to the VHB membrane; 2. We cut the textile (ElectroLycra or Conductive-Knit) into two circular electrode patterns using the Cricut cutter, as shown in Figure 4A. Both patterns had an outer dimeter of 50 mm, and one had an inner diameter of 3 mm, whereas the other had an inner diameter of 16 mm. This prevented dielectric breakdown between the top and bottom electrodes; 3. We bonded the textile electrodes onto the centre of the VHB membrane. A 3 mm diameter hole was cut in the centre of the electrode through the VHB membrane to enable the device to be breathable; 4. We cut out a 51 mm diameter circle of cellulose sheet (Q-CONNECT, 0.1 mm thickness) and cut three straight lines as show in Figure 4B to make the auxetic structure. We then bonded the edge of the auxetic structure onto the textile electrodes using the Sil-poxy glue (cure time: 12 min, Bentley Advanced Materials, Kidderminster, Worcestershire, UK), forming the final monolithic breathability control device. A schematic diagram of the working principle of the DEA driven breathability control device is presented in Figure 5A. 

When no voltage is applied, the auxetic structure is closed, and no air can pass through the hole in the membrane. When a voltage of 6 kV is applied for 10 s, the central DEA formed by the two textile electrodes and the VHB membrane expands. This forces the auxetic structure to expand, causing it to open at the centre in a slit. This opening allows air from below the membrane to pass through the hole to the top of the device. The opening is reminiscent of the turgor-driven actuation of leaf stomata. We fabricated one device using ElectroLycra and another using Conductive-knit. Their dynamic breathability, as demonstrated by the area changes in the central opening, is shown in Figure 5B. 

**Figure 6:**
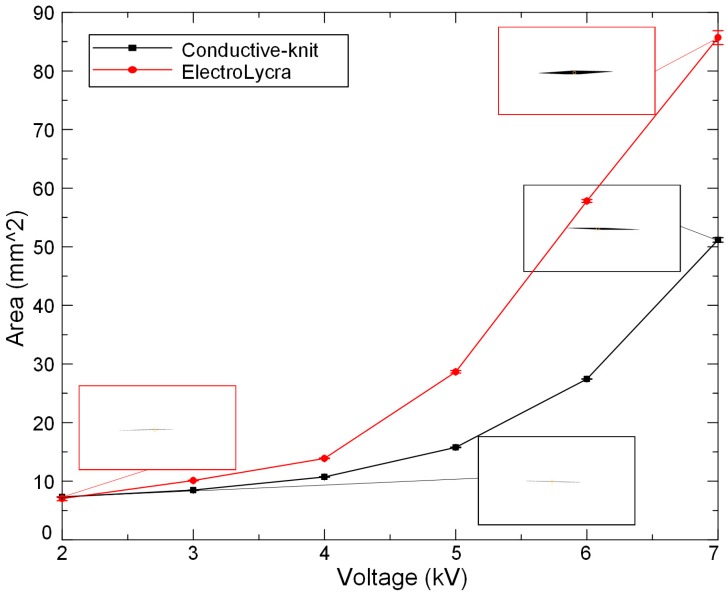
Breathability control comparison between the ElectroLycra and Conductive-knit textile DEAs from 2 to 7 kV. Area denotes the area of the aperture slit in the auxetic (images show slit at minimum and maximum voltages).

Here we see a larger response from the ElectroLycra electrodes. This is because ElectroLycra has almost uniform strain in both the *x* and *y* axes, whereas Conductive-knit exhibits large strain in one axis only. This means that the ElectroLycra textile enabled two directional actuations of the auxetic structure whereas Conductive-knit only enabled one. For both devices, as the applied voltage is increased from 2 to 7 kV, the overall device breathability increases, as shown in Figure 6. At each voltage, the dynamic area change of the auxetic structure due to the DEA actuation was captured by the 50 fps Panasonic DMC-G80 camera. Each video was firstly processed into individual images, which were then de-noised. A mask was used to extract the regions of interest, a 7 mm × 25 mm rectangular area in the middle of the auxetic structure, which was then thresholded on intensity value to yield a binary image. The breathability area was finally calculated as the sum of the black pixels, as shown in Figure 6.

### 3.2. 3D Kirigami Thermal Regulation Device

**Figure 7:**
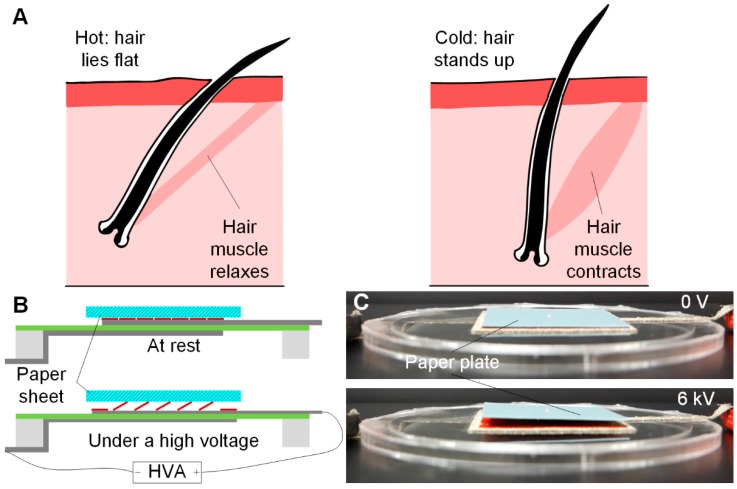
Bio-inspired e-textile DEA driven skin thermal regulation device working principle and prototypes. (**A**) Schematic diagram of the hair thermal regulation principle. (**B**) Schematic diagram of the E-textile DEA driven skin thermal regulation device working principle at rest and under a high voltage. (**C**) Prototype device at 0 V and 6 kV.

The transition from 2D to 3D is attractive when combined with actuators such as DEA, which inherently operate in a 2D plane. Here we take inspiration from the ability of animal hairs [29], including human [30], to transition from a state where they lay flat against the (2D) skin to an erectile state where they stand perpendicular to the skin (3D). This hair erection is driven by small muscles within each hair follicle. The ability to erect the hairs enables an animal to trap air in its hair or fur and thereby to control its heat loss and regulate its internal temperature. As shown in Figure 7A, in hot weather, the hair lies flat as the hair muscle is relaxing, so less air is trapped close to the skin. More heat is therefore lost by radiation. In cold weather, in contrast, the hair erector muscle contracts and the hair stands up, thereby trapping a thicker layer of air to prevent heat loss. Our goal here is to replicate this process to control the amount of air that is trapped against the skin. To achieve this, we fixed the ends of a planar kirigami structure (made from cutting a 0.2 mm high-density polythylene film) to a square (55 mm × 55 mm) Conductive-knit textile DEA to form a monolithic bio-inspired 3D e-textile thermal regulation device. Figure 7B shows the operating principle and Figure 7C shows the prototype device. When activated, the DEA actuator stretches the kirigami structure uniaxially. This caused the planar beams within the structure to buckle into a 3D configuration, forcing the top surface of the structure upwards and trapping air within it (see Figure 7B). Please see the demonstration of this in the Supplementary Video S1.

**Figure 8:**
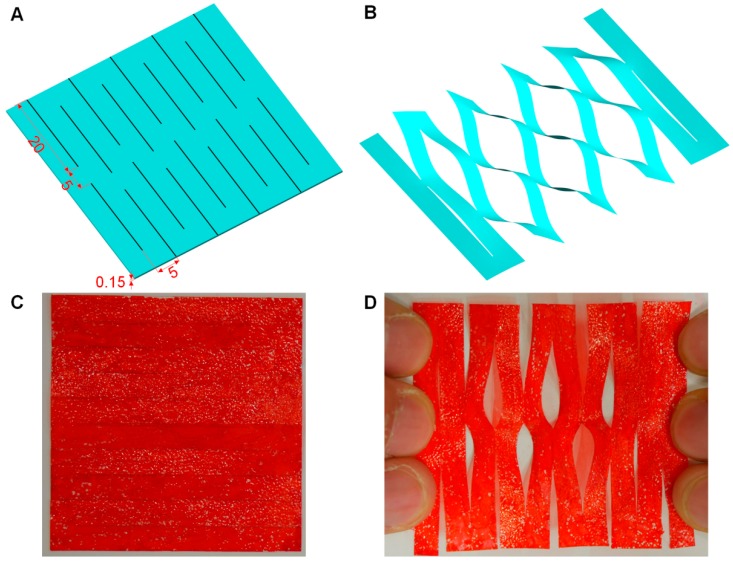
Kirigami structural design and prototype. (**A**) Design parameters for the kirigami structure. (**B**) Deformation into 3D (FEA model). (**C**) The prototype of the kirigami structure at rest state. (**D**) The prototype of the kirigami structure stretched by hand (The clear kirigami film is coloured red for clarity).

**Figure 9:**
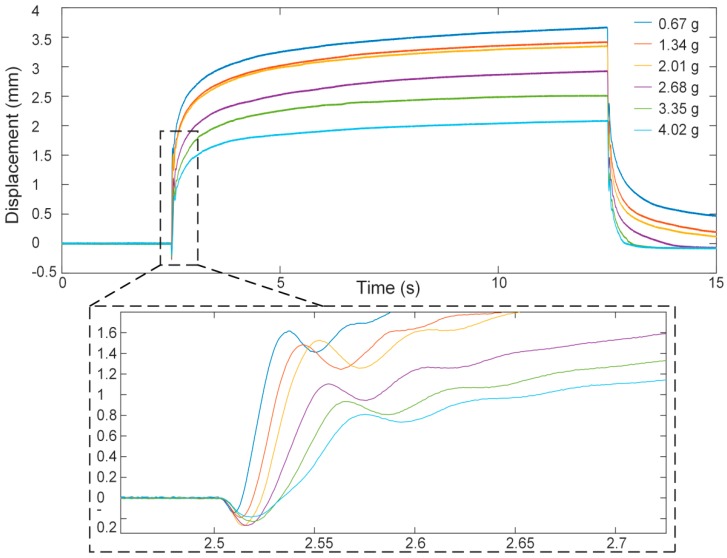
Vertical displacements of different loads when a 6 kV step input was applied to the e-textile DEA driven skin thermal regulation device.

The un-stretched kirigami structure is show in Figure 8A, labelled with the principle design parameters. Figure 8B shows the deformation of the kirigami structure into 3D as it is stretched (in an Ansys FEA model). Figure 8C,D show the prototype of the kirigami structure in the rest state (C) and stretched by hand (D). To test the effectiveness of the 2D-to-3D actuation under different loading conditions, we placed different numbers of paper sheets (50 mm × 50 mm, each 0.67 g) on top of the kirigami structure as it actuated. A laser displacement sensor (LK-G3001, Keyence, Osaka, Japan) was used to record the vertical displacements of the loads, as shown in Figure 9. Vertical displacement, at an applied voltage of 6 kV, decreased with increasing loads. The maximum vertical displacements against different loads (after applying voltage for 10 s) is summarized in Table 2. Under a load of 0.67 g, a vertical displacement of 3.66 mm was achieved.

It is also interesting to note the dynamic response of the device as a step voltage is applied (as shown in the zoomed graph in Figure 9). When applying 6 kV to the DEA, the vertical displacements of all loads initially decreased before sharply increasing and overshooting (after approx. 100 ms), followed by a smooth exponential rise to the final height. The initial decrease of the vertical displacements is attributed to the rapid deformation of the DEA and its relaxation downwards due to gravity. Simultaneously the kirigami structure undergoes increasing axial (end-to-end) stress. At some stress thresholds, the kirigami structure suddenly buckles out-of-plane, rapidly stretching and increasing in thickness. This buckling is of sufficient speed to accelerate the load upward and cause an overshoot. A subsequent increase in DEA actuation causes the already-buckled kirigami structure to slowly stretch and increase in thickness, and for the load to increase in height. 

**Figure 10:**
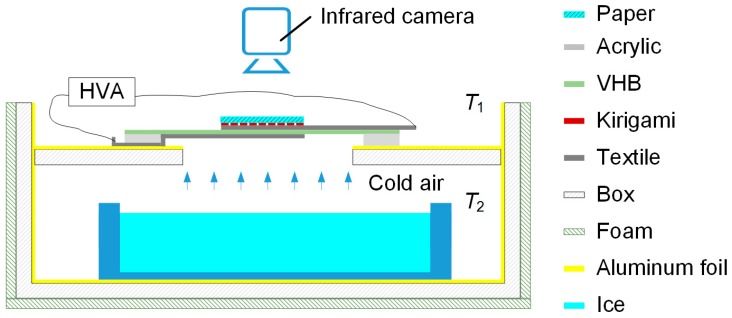
Schematic diagram of the temperature regulation test rig.

**Figure 11:**
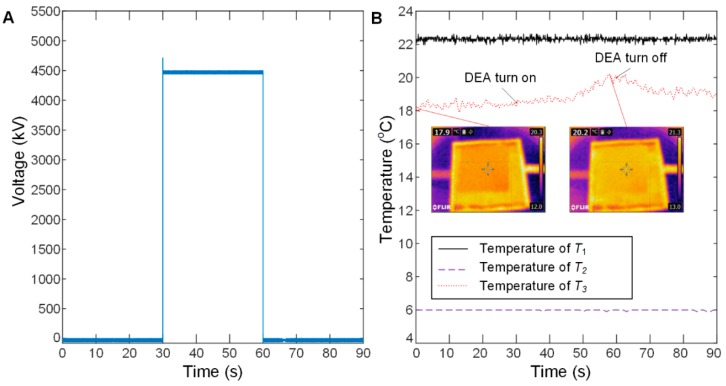
(**A**) The voltage signal driving the DEA thermal regulation device. (**B**) The temperature of the chambers and the paper as the DEA is actuated.

In order to further test the 3D kirigami thermal regulation device, a test platform was set up, as shown in Figure 10. An acrylic box with two chambers, arranged one above the other, was fabricated by laser cutting sheets and bonding them together. A circular orifice was cut in the partition separating the chambers, and the top chamber was open to the air, thereby acting as a room-temperature buffer. The box was thermally insulated with aluminium foil and insulating foam (approx. 10 mm thickness). A tank with ice was placed in the lower chamber. The 3D kirigami thermal regulation device was placed at the bottom of the upper chamber to completely cover the circular orifice in the partition. A piece of paper (38 mm × 38 mm, 0.089 g) was attached to the top of the kirigami structure to act as a temperature indicator. The foil and foam insulation kept the bottom chamber at a constant low temperature for the duration of the experiment. The temperatures of the top and bottom chambers were captured by a digital temperature sensor (LM35, Texas Instruments, Dallas, TX, USA) and a digital thermocouple (USB-TC01, National Instruments, Austin, TX, USA), respectively. An infrared camera (FLIR E4, FLIR^®^ Systems, Inc., Wilsonville, OR, USA) was placed above the upper chamber to capture the temperature change of the paper. *T*_1_, *T*_2_ and *T*_3_ denote the temperatures of the top chamber, the bottom chamber and the paper surface, respectively. The DEA was actuated using the voltage input shown in Figure 11A, where no voltage was applied to the DEA for 30 s and then 4.5 kV was applied to the DEA for 30 s and finally no voltage was applied for 30 s. As can be seen in Figure 11B, the temperature of the upper chamber, *T*_1_, was kept constant during the experiment at approximately 22.1 °C. *T*_2_, the temperature of the lower chamber, was kept at a constant temperature of approximately 6 °C. When the DEA was not actuated, *T*_3_, the temperature of the paper at the interface between the chambers, remained approximately the same, with a very small steady rise as heat energy slowly moved from the upper chamber to the lower chamber. This heat flow was not sufficient to significantly raise *T*_2_, because the lower chamber had a much higher thermal inertia than the DEA and indicator paper. When the DEA was not actuated, the paper was in close contact with the flat kirigami structure, which in turn contacted the fabric electrode of the DEA. This resulted in a conduction path from the top chamber to the bottom and the low temperature of the indicator paper. When the DEA was actuated, the bending of the kirigami structure from 2D to 3D forced the paper away from this conductive contact, creating an insulating air gap between the paper and the DEA. This caused the indicator paper to increase in temperature towards *T*_1_, the upper chamber temperature, as clearly shown in Figure 11B. The temperature of the centre of the paper increased from 17.9 to 20.2 °C, and the mean temperature of the paper increased from 20.3 to 21.3 °C. After the DEA was turned off, the paper returned to close contact with the DEA structure and started to cool. At the end of the experiment, the centre of the paper had fallen to just below 19 °C. Actuation of the thermal regulation device resulted in a temperature change at the centre of the paper of 2.3 °C and across the sheet of 1 °C, showing proof-of-concept for a wearable thermal regulation device. Figure 11B shows two thermal images of the paper, one before the DEA actuation and one showing the highest temperature during DEA actuation. 

## 4. Conclusions and Future Work

Conductive textiles are more comfortable electrode materials than potentially carcinogenic carbon grease and nano-particle electrodes, are low cost and readily available. They yield the opportunity to develop simple, comfortable and wearable soft robotic devices, and complete soft robots. The key findings of this work include: (1) Conductive-knit textiles are more effective for unidirectional DEA applications as evidenced by their large anisotropy (a relative strain difference of 150% in principle axes), whereas ElectroLycra is more suited to bidirectional DEA applications due to high isotropy (a relative strain difference of 11.1% in principle axes); (2) We demonstrated a 2D e-textile DEA-driven skin breathability control device and a bio-inspired 3D DEA-driven kirigami thermal regulation device. When increasing the applied voltage from 2 to 7 kV, the breathability increased approximately quadratically. The thermal regulation device was shown to have the ability to change temperature as shown in Figure 11B.

The contributions of this work include: (1) A quantitative evaluation of five different stretchable conductive textiles for DEA actuation applications; (2) the development and demonstration of a 2D e-textile DEA-driven active breathability control device and (3) the development and demonstration of a bioinspired 3D e-textile kirigami active thermal control device. Future work will include the development of e-textile dielectric elastomer sensors and generators, e-textile wearable and adaptive compression systems for the human body and autonomous e-textile soft robots.

## Figures and Tables

**Table 1:** Mechanical and electrical properties of the five different conductive and stretchable textiles.

Fabric Name	Thickness [mm]	Mass [g/m^2^]	Resistance [Ω]	Construction Method
EeonTex ^1^	0.36 ± 0.017	175.0	60,0667 ± 3091	Warp knit
Softmesh ^2^	0.21 ± 0.008	31.3	20.20 ± 0.70	Knit
Knit Jersey ^3^	0.61 ± 0.014	162.5	21.67 ± 0.24	Knit
Conductive-knit ^4^	0.52 ± 0.005	193.8	23.23 ± 0.60	Knit
ElectroLycra ^5^	0.49 ± 0.021	150.0	21.87 ± 0.26	Knit (medical grade)

^1^ EconTex was purchased from www.sparkfun.com/products/retired/14112, and it is made of 72% nylon and 28% spandex and coated with a proprietary conductive formulation, wales 38–44, courses 50–56; ^2^ Softmesh as purchased from mindsetsonline.co.uk/shop/softmesh/, and it is made of stretchy silver coated sheer nylon weave; ^3^ Knit Jersey was purchased from www.adafruit.com/product/1364, and it is made of 63% cotton, 35% silver yarn and 2% spandex; ^4^ Conductive-knit was purchased from www.kitronik.co.uk/2717-conductive-fabric-stretch.html, and it is made of 94% nylon and 6% elastomer and plated silver; ^5^ ElectroLycycra was purchased from mindsetsonline.co.uk/shop/electrolycra/, and it is made of 76% nylon and 24% elastic fiber fabric and plated with silver.

**Table 2:** Maximum vertical displacements against different loads.

Loads [g]	Maximum Vertical Displacements [mm]
0.67	3.66
1.34	3.42
2.01	3.34
2.68	2.92
3.35	2.51
4.02	2.10

## Supplementary Materials

The following are available online at https://www.mdpi.com/2073-4360/11/7/1199/s1, Video S1: Demonstration of E-textile DEA driven breathability and thermal control devices, Supp_Data1.
